# RETRACTION: Synergistic Effect of Zuo Jin Wan on DDP-Induced Apoptosis in Human Gastric Cancer SGC-7901/DDP Cells

**DOI:** 10.1155/ecam/9793487

**Published:** 2025-06-17

**Authors:** Evidence-Based Complementary and Alternative Medicine

RETRACTION: Q.-F. Tang, Q. Ji, Y.-Y. Qiu, et al., “Synergistic Effect of Zuo Jin Wan on DDP-Induced Apoptosis in Human Gastric Cancer SGC-7901/DDP Cells,” *Evidence-Based Complementary and Alternative Medicine* 2014, no. 1 (2014): 1–10, https://doi.org/10.1155/2014/724764.

The above article, published online on 3 March 2014 in Wiley Online Library (https://wileyonlinelibrary.com), has been retracted by John Wiley & Sons Ltd.

The retraction has been agreed following an investigation of the concerns raised by *Actinopolyspora biskrensis* on PubPeer [1], which identified an instance of figure duplication.

Specifically:

- Figure 6: The images of the Bax expression in DDP + ZJW (H-dose) cell colony and Bcl-2 expression in SGC-7901/DDP cells are images of the same tissue.

As a result of the investigation, the data and conclusions of this article are considered unreliable.

The authors were informed of the decision to retract the article but did not provide a response.
